# Orbitofrontal cortex and amygdala as key regions for the classification of small animal phobia: A structural MRI machine learning study

**DOI:** 10.3758/s13415-026-01459-5

**Published:** 2026-06-01

**Authors:** Alessandro Grecucci, Alessandro Scarano, Ascensión Fumero, Luca Sarzi Maddidini, Enrico Ruà, Francisco Rivero, Rosario J. Marrero, Teresa Olivares, Yolanda Álvarez-Pérez, Wenceslao Peñate

**Affiliations:** 1 Clinical and Affective Neuroscience Lab - Cli.A.N. Lab, Department of Education, Psychology and Communication Sciences, University of Bari, Bari, Italy; 2 Department of Psychology and Cognitive Science, University of Trento, Trento, Italy; 3 Departamento de Psicología Clínica, Psicobiología y Metodología, Facultad de Psicología, Universidad de La Laguna, 38200 La Laguna, Tenerife Spain; 4 Instituto Universitario de Neurociencia (IUNE), Universidad de La Laguna, 38200 La Laguna, Tenerife Spain; 5 Facultad de Ciencias de La Salud, Universidad Europea de Canarias, 1 38300, La Orotava, Tenerife Spain; 6 Fundación Canaria Instituto de Investigación Sanitaria de Canarias (FIISC), 35012 Las Palmas de Gran Canaria, Las Palmas Spain

**Keywords:** Animal phobia, Machine learning, Clinical neuroscience, Amygdala, Orbitofrontal cortex

## Abstract

Small animal phobia (SAP) is an anxiety disorder characterized by an intense fear triggered by small animals. Existing studies on SAP have primarily used univariate analyses and small, unbalanced samples, leading to inconsistent findings. Many brain structures have been proposed as potential candidates for encoding SAP, including the amygdala, the orbitofrontal cortex, the insula, the cingulate gyrus, the hippocampus, the precuneus, and the putamen. However, no previous study has systematically compared the predictive role of each of these regions to determine which one provides the most accurate classification of individuals with SAP. To address this question, we tested the predictive power of these brain regions associated with SAP. Structural MRI images of 32 individuals with SAP and 90 matched healthy controls were analyzed by using multiple Binary Support Vector Machines (BSVM). Our results showed that the most critical regions for classifying SAP, in order of significance, were the orbitofrontal cortex (balanced accuracy = 80.83%), the amygdala (76.15%), the insula (71.35%), and the posterior cingulate (71.25%). However, only the orbitofrontal cortex and the amygdala consistently exceeded chance levels across all additional parameters considered (precision, specificity, sensitivity, and F1-score). In conclusion, this study enhances the understanding of the neural basis of small animal phobia, suggesting new research directions and diagnostic strategies using advanced machine learning methods applied to neuroimaging data. Clinical implications are also discussed.

## Introduction

Anxiety disorders, including generalized anxiety disorder, panic disorder, social anxiety disorder, and specific phobias, are some of the most prevalent mental health conditions globally (Santomauro et al., 2021). These disorders are marked by excessive and persistent anxiety that disrupts daily functioning, often leading to avoidance behaviors, social distress, and occupational impairment (Craske et al., 2011; Mah et al., 2016; Olatunji, 2019). Specific phobias (SP), a subcategory of anxiety disorders, involve intense, irrational fears triggered by specific stimuli or situations and have a prevalence rate of about 10% in the general population (Fyer, 1998). Among these, small animal phobia (SAP)—which includes fears of insects, spiders, reptiles, and rats—remains under-researched, and its neural underpinnings are still not fully understood.

Functional neuroimaging studies consistently reveal that limbic and paralimbic regions, such as the amygdala, insula, and cingulate cortex, are activated in response to phobic stimuli (Del Casale et al., 2012; Peñate et al., 2017; Wright et al., 2003). Additionally, brain areas outside the limbic system, including the prefrontal and orbitofrontal cortices, also show altered activation and volume in phobic individuals. Structural neuroimaging studies support these findings, showing differences in gray and white matter between those with specific phobias and healthy controls.

For instance, an early study found increased cortical thickness in individuals with animal phobias (AP), particularly in the bilateral insular cortex, anterior and posterior cingulate cortex, occipital cortex, and occipitotemporal cortex (Rauch et al., 2004). Subsequent research has highlighted the role of the right anterior insula in anxiety sensitivity (AS), showing that SAP individuals with higher AS also have greater insular cortex thickness and volume, suggesting that this region plays a key role in mediating heightened anxiety (Rosso et al., 2010). In another study, SAP individuals displayed a 13% increase in left amygdala volume compared with healthy controls (Fisler et al., 2013).

Hilbert et al. (2015) expanded this research by investigating various phobias (e.g., dental and snake phobias). They found increased gray matter volumes in the right subgenual anterior cingulate cortex (ACC), left medial orbitofrontal cortex (OFC), precuneus, calcarine sulcus, fusiform gyrus, and vermis. These findings were consistent across different phobia subtypes. More recent studies on SAP also found decreased gray matter volumes in the insula, OFC, superior medial frontal cortex, and anterior cingulate cortex while showing increased gray matter volume in the left putamen (Rivero et al., 2023).

These findings provide crucial insights into the structural and functional alterations associated with small animal phobia, but further research is necessary to fully understand the brain circuits involved. Indeed, despite the valuable findings of prior studies, research investigating the neurological basis of anxiety disorders, particularly phobias such as small animal phobia (SAP), has faced significant challenges that limit the depth and generalizability of their conclusions. One major issue is the frequent reliance on small sample sizes (Rauch et al., 2004; Rivero et al., 2023; Wright et al., 2003), which raises concerns about the robustness and replicability of results. Additionally, gender imbalances in participant samples—often skewed toward female subjects—have been noted. This bias is partly driven by the higher prevalence of animal phobias in women compared with men, with a ratio estimated to be as high as 3:1 (Kendler et al., 2001). While this gender discrepancy reflects the real-world prevalence of SAP, it may inadvertently influence neural findings, making them less generalizable across genders. Another critical limitation of past research is the widespread use of mass univariate analyses, which examine individual voxels independently, overlooking the complex statistical interdependencies between them (Grecucci et al., 2022, 2023; Sorella et al., 2019). Moreover, the generalizability of these findings and their ability to predict new cases (e.g., as potential biomarkers) have rarely been assessed (Sorella et al., 2019), further limiting their clinical applicability.

These methodological limitations often lead to inconsistent and inconclusive results. For example, while regions like the orbitofrontal cortex and amygdala are well-established in the literature for their roles in fear conditioning and emotional processing, their involvement in SAP remains uncertain. Other regions, such as the cingulate, the insula, the hippocampus, the precuneus, the hypothalamus, and the putamen, have been inconsistently reported. Further research is necessary to assess the specific contribution of each of these regions, and to detect the most relevant ones.

The aim of this study is to compare the main regions associated with SAP and to assess for the first time their relative contribution to classify individuals with SAP. Moreover, to overcome the limitations of the statistical methods used in the vast majority of the previous studies, we aim to use a multivoxel pattern analysis, also known as supervised machine learning (ML), capable of detecting complex patterns in brain imaging data, extract a predictive model and test for its generalizability (Norman et al., 2006). In other words, these models can be used as predictive models to classify never seen before individuals passing their brain features trough the statistical model. Machine learning methods have been successfully used in the classification of various psychiatric and neurological conditions, achieving accuracy rates between 60 and 90% across disorders, such as depression, schizophrenia, borderline personality disorder, narcissistic personality disorder, and social anxiety disorder (Grecucci et al., 2022, 2023; Jornkokgoud et al., 2023; Mwangi et al., 2012; Sorella et al., 2019).

A few recent attempts to apply ML methods in the context of phobia have been reported. In a recent study, Böhnlein & colleagues (2021) applied ML techniques in a functional MRI study to classify spider phobic individuals with 73% balanced accuracy based on their neural responses to emotional faces. Key regions involved included the inferior parietal cortex, fusiform gyrus, middle cingulate, postcentral cortex, and insula. In a more recent study, Scarano & colleagues (under review) reported that a brain network including, among others, regions, such as the temporal pole, the frontal cortex, the amygdala, and the insula were predicting the diagnosis of SAP. In another study, Lueken et al. (2015) used a Gaussian process classifier to distinguish between individuals with spider and dental phobias and healthy controls, achieving 89% accuracy for the spider phobia group. Brain regions, such as the orbitofrontal cortex, insula, cingulate, and hippocampus, contributed to this classification accuracy.

Overall, these studies have mainly focused on specific phobias like spider phobia, leaving the broader category of small animal phobia, including phobias of insects, reptiles, and other animals, relatively unexplored. Besides they tested whole brain data without assessing the specific role of each brain region. These gaps in the literature underscores the need for further research using ML techniques to investigate the neural mechanisms underlying SAP. By testing specific brain regions and assessing their contribution to classifying SAP, we aim to expand our knowledge of the brain structures underlying phobias.

We hypothesize that the brain structures previously reported in SAP literature and considered in the present study (the amygdala, the orbitofrontal cortex, the insula, the cingulate, the hippocampus, the precuneus, and the putamen) may all play a role in classifying SAP (Rivero et al., 2023; Rondina et al., 2018). However, we expect the maximum information to be included in the amygdala and the orbitofrontal cortex for their role in fear conditioning, extinction, anxiety, and fear related response (Del Casale et al., 2012; Peñate et al., 2017; Wright et al., 2003).

## Methods

### Participants

The Small Animal Phobia (SAP) group included 32 individuals, of which 25 were females, with a mean age of 34.4 years (± 11.07). They received a diagnosis for Specific phobia of small animals (insects, spiders, reptiles, rodents) as determined by the Composite International Diagnostic Interview (CIDI), Version 2.1 (Kessler & Üstün, 2004). Additional Criteria: Participants had no visual problems, and their specific phobia was the primary psychological condition without influence from other health disorders. The Control Group consisted of 90 healthy individuals, of which 59 were females, with a mean age of 31.95 years (± 10.25). The control group comprised 32 subjects acquired in the previous study (Rivero et al., 2023), plus 58 subjects derived from the UCLA Consortium for Neuropsychiatric Phenomics dataset (OpenNeuro, Accession No. ds000030) (Gorgolewski et al., 2017). The control subjects from the UCLA dataset were chosen to match the SAP group regarding gender and age and were obtained through community advertisements, clinical outreach, and online portals. The inclusion criteria for both datasets required participants to have at least 8 years of formal education and be proficient in either English or Spanish.

All control subjects had no history of psychiatric or neurological disorders, similar to the SAP group in terms of education and language proficiency. All participants were right-handed (Table 1). Anxiety severity was assessed by using the Hamilton Anxiety Rating Scale (HARS; Hamilton, 1959) and the Beck Anxiety Inventory (BAI) (Beck et al., 1988). In addition, participants completed the Situation–Response (S–R) Inventory of Anxiousness (Endler et al., 1962), which measures cognitive, physiological, and behavioral responses to anxiety-provoking situations, including phobia-related stimuli.

**Table 1:** Demographic and diagnostic information about the participants, including their number, gender distribution, mean age, and inclusion criteria

	SAP (n = 32)	CTRL (n = 90)	*p*
n	32	90	
Sex	7 M, 25F	31 M, 59F	0.19
Age	Mean age = 34.4 (SD ± 11.07)	Mean age = 31.95 (SD ± 10.25)	0.18
Inclusion criteria	Smal animal phobia diagnosis, Right-handedness, no contraindications for MRI scanning	No history of psychiatric or neurological disorder, right-handedness, no contraindications for MRI scanning	

### MRI data acquisition

MRI data were collected from two different scanners to include participants from both the SAP and control groups. The SAP group and 32 control participants were scanned using a GE 3 T Sigma Excite HD system, while additional control subjects were scanned on a 3 T Siemens Trio scanner. Participants scanned on the GE 3 T Sigma Excite HD were instructed to keep their eyes closed, stay relaxed without falling asleep, and remain as still as possible during the session. High-resolution, three-dimensional T1-weighted images were acquired with specific parameters, including a repetition time (TR) of 8,852 ms, an echo time (TE) of 1,756 ms, and a flip angle of 10°. The images consisted of 172 sagittal slices, each 1 mm thick, with a field of view of 256 mm^2^ and a voxel size of 1 mm^3^. A neuroradiologist reviewed all scans to ensure no visible movement artifacts or gross structural abnormalities were present. A similar high-resolution T1-weighted anatomical scan was obtained using the MPRAGE sequence for the Siemens Trio scanner for the additional 58 control participants. The acquisition parameters slightly differed, with a TR of 1.9 s and a TE of 2.26 ms, along with 176 slices and a thickness of 1 mm. The matrix size was set to 256 × 256, and the field of view was 250 mm^2^, ensuring high image quality and resolution.

### Preprocessing

Before analyzing the data, a thorough preprocessing routine was performed to prepare the MRI scans for further examination. This process utilized SPM12 software and the Computational Anatomy Toolbox (CAT12), which runs in MATLAB. The data preprocessing was designed to eliminate artifacts and segment different brain tissues accurately. The first step involved segmenting each participant’s brain into gray matter, white matter, and cerebrospinal fluid. After segmentation, a technique known as modulated normalized writing was applied to account for any potential volume differences that may have arisen during the segmentation process. For the registration of the images, the study employed Diffeomorphic Anatomical Registration through Exponential Lie Algebra (DARTEL), which is known for being a more accurate method of aligning whole-brain images compared to more traditional approaches (Grecucci et al., 2016; Pappaianni et al., 2018; Yassa & Stark, 2009). After registration, the images were normalized to the MNI space, a standardized brain template commonly used in neuroimaging research. Once the images were normalized, they were spatially smoothed to ensure that any slight differences in individual brain anatomy would not affect the results. A Gaussian smoothing kernel with a full width at half maximum (FWHM) of 12 mm was applied to the data, which is a common practice in multivariate pattern analysis (MVPA) of structural brain images (Monté-Rubio et al., 2018). Because participants were scanned on two different MRI scanners, it was necessary to ensure that the data from the two scanners were comparable and not influenced by scanner-specific noise. To address, this issue, we applied Independent Component Analysis (ICA) (McKneown et al., 1998), a method that helps identify and separate any potential noise source from the true effect within the data. ICA detects and reconstructs the signal coming from different sources in the gray matter images, identifying independent clusters of voxels covariant along the entire sample (Xu et al., 2009).

ICA was performed on all the scans inside the GIFT toolbox (http://mialab.mrn.org/software/gift/). The minimum description length (MDL) criterion identified 13 distinct components within the data. Statistical analyses indicated that one component—IC13—significantly differed between the two scanners (t(1,120) = 4.191, *p* < 0.001) and not with being a phobic individual or a control. To minimize any bias introduced by this neural component, we removed the effect of IC13 from the data in the subsequent analyses. Specifically, a mask of the IC13 component including the affected voxels associated with the scanner-specific noise was created. This mask was then combined with the SPM_noeyes.nii mask used inside the toolbox PRONTO for ML analyses to exclude any potential scanner artifact. The ImCalculator tool in SPM12, was used to this aim. This mask was then applied to our data to remove any voxels associated with the scanner noise (IC13) and any additional artifact (SPM_noeyes.nii) from the subsequent ML analyses. Component IC13 was classified as artefactual based on established criteria, including its spatial distribution (predominantly outside grey matter), edge-related patterns, and high-frequency temporal characteristics (Griffanti et al., 2017; Pruim et al., 2015). Component IC13 was classified as artefactual based on established criteria including spatial distribution (predominantly outside grey matter), edge-related patterns, and high-frequency temporal characteristics (Griffanti et al., 2017; Pruim et al., 2015). To further ensure that its removal did not affect biologically meaningful signal, we examined its overlap with grey matter ROIs, confirming minimal contribution to canonical grey matter networks. We quantified its overlap with a grey matter mask, showing that only 12% of its voxels were located within grey matter. This limited overlap supports its classification as a noise-related component. Scanner/site was included as a covariate (regressor of no interest) in all group-level analyses and model validation procedures to account for inter-scanner variability (Fortin et al., 2018; Yamashita et al., 2019).

### Data analysis

To differentiate individuals with small animal phobia (SAP) from healthy controls based on brain imaging data, we employed a Binary Support Vector Machine (BSVM) approach using the Pattern Recognition for Neuroimaging Toolbox (PRoNTo) (Schrouff et al., 2013). BSVMs are specifically designed for binary classification tasks; they find the optimal hyperplane that maximizes the margin between two classes, enhancing generalization to new data. This method is well-suited for neuroimaging studies where distinguishing between conditions based on complex brain features is crucial. By leveraging the BSVM approach, we aimed to capture the subtle and complex patterns in gray matter density that differentiate SAP individuals from controls. The use of SVMs in neuroimaging allows for handling high-dimensional data and mitigates overfitting through regularization, making it a robust choice for our classification task.

### Preprocessing and feature extraction

We started by preprocessing the gray matter images of both groups. This included applying a denoising mask designed to reduce scanner-related noise, as our participants were scanned on two different MRI machines. Then, specific masks for each region (amygdala, orbitofrontal cortex, insula, cingulate, hippocampus, precuneus, and putamen) were applied, and the gray matter density (GMD) values of the voxels inside each structure considered were extracted.

The GMD values were then reorganized into a data matrix, structured as Nsamples × Nfeatures. This matrix was then used to compute a similarity matrix or kernel (Hofmann et al., 2008), which represents the relationships between participants. This kernel, of size Nsamples × Nsamples, was fed into the BSVM algorithm.

### Model training

We trained separate BSVM classifiers for each region to distinguish between the 32 SAP participants and the 90 matched controls based on regional GMD patterns. We optimized the model's soft-margin hyperparameter C using a nested cross-validation scheme. Hyperparameter values tested included 0.0001, 0.01, 1, 10, 100, and 1,000 (Claesen & De Moor, 2015).

This cross-validation process used two loops: the inner loop for optimizing hyperparameters and the outer loop for evaluating model performance. In the outer loop, we used the one-leave-out subjects cross-validation technique. Each subject served as a test subject once, while the other subjects were used for training. This ensured that each participant contributed to both training and testing, preventing overfitting, and ensuring that the test set remained independent. Using this strategy, we assessed the model’s accuracy, which we measured through balanced accuracy—a crucial metric when dealing with imbalanced datasets like ours, where there were more control subjects than SAP participants. In addition to balanced accuracy, we computed other performance metrics, including: F1-Score: A metric that balances precision and recall, useful when the distribution between classes is uneven; Sensitivity (Recall): The model's ability to correctly identify SAP individuals; Specificity: The model's ability to correctly classify controls. These metrics provided a comprehensive understanding of how well the model could differentiate between the two groups. To confirm the statistical significance of our classification results, we conducted 5,000 permutation tests for each model. This involved randomly permuting the labels (SAP and control) and rerunning the analysis to generate a p-value for the model’s performance. This method adds statistical rigor to the results, ensuring that our findings were not due to chance.

**Fig. 1:**
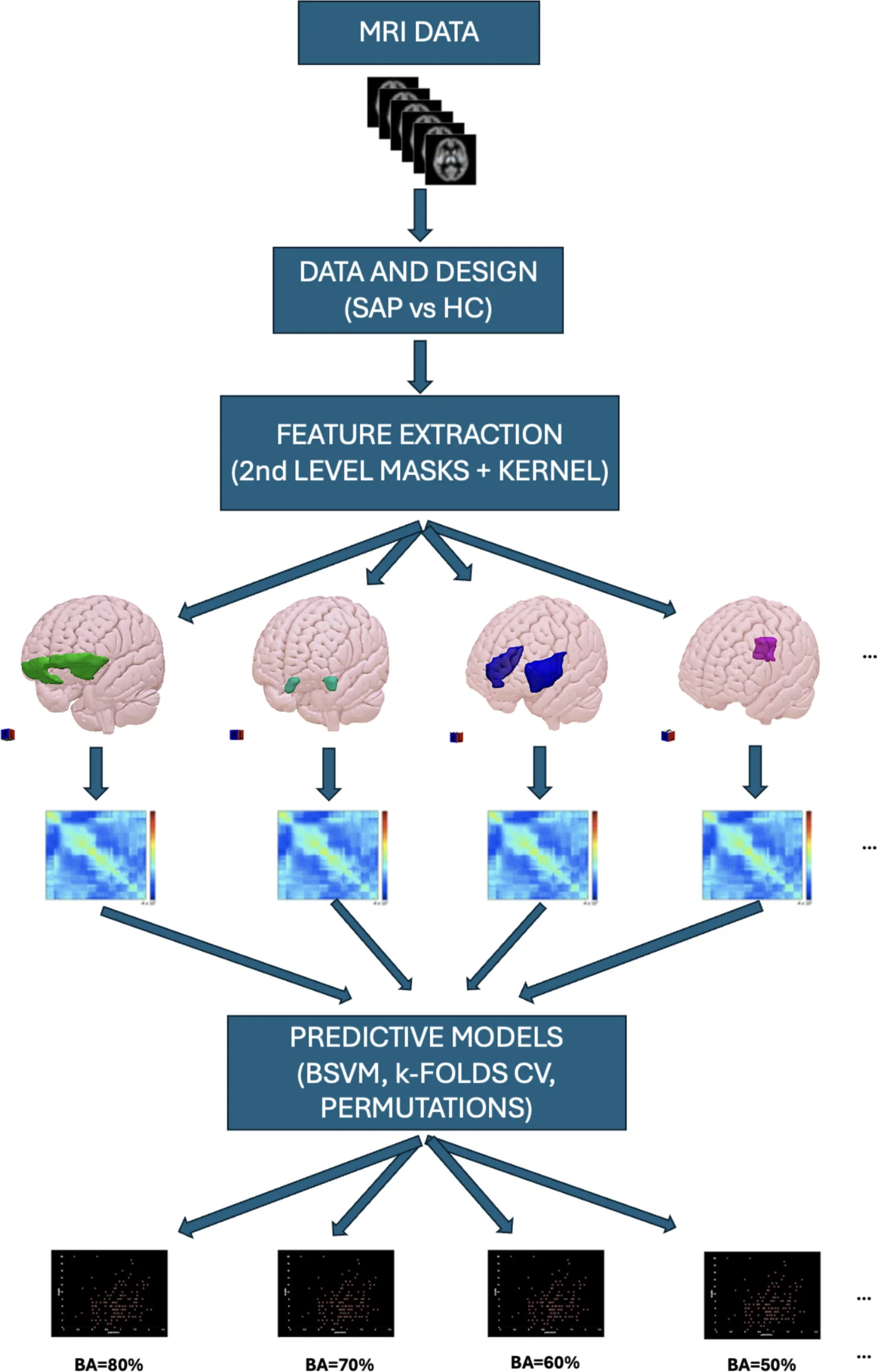
Graphical representation of the method. MRI images of SAP and HC were considered. GMC values were extracted from the voxels inside each region. These values were reorganized in kernel matrices. A BSVM algorithm was then trained in a fivefold cross-validation sequence. Permutations were then used to assess the statistical reliability of each model

To account for differences in voxel counts across regions, we normalized the features and performed mean centering to ensure comparability. This allowed us to test each network's classification accuracy, exploring its potential role in distinguishing SAP individuals from controls (Fig. 1). Although the present analysis focused on regional classification performance, linear SVM models allow inspection of voxel-wise weight maps, which reflect the contribution of individual features to the decision boundary. These maps can provide qualitative insights into subregional contributions within each ROI.

## Results

### Demographic results

No significant age difference was found between the SAP and control groups [t = 1.33, *p* = 0.186]. No significant gender difference was observed between the groups [t = 1.317, *p* = 0.19].

### Predictive models

We applied a Bonferroni correction to account for multiple comparisons, resulting in a significance threshold of *p* = 0.05/(2 groups × 10 models) = 0.0025. The analyses conducted using a Binary Support Vector Machine (BSVM) highlighted a fundamental role in specific phobias of the amygdala, orbitofrontal cortex, insula, and posterior cingulate cortex compared with the other areas examined. In order of significance: orbital frontal cortex (BA: 80.83%; *p*-value: 0.0020; AUC: 0.91), amygdala (BA: 76.15%; *p*-value: 0.002 AUC: 0.86), insula (BA: 71.35%; *p*-value: 0.002; AUC: 0.85) and posterior cingulate cortex (BA: 71.25%; *p*-value: 0.002; AUC: 0.8) exceeded the Bonferroni corrected significance threshold (*p* = 0.0025), validating their discriminative power.

**Fig. 2:**
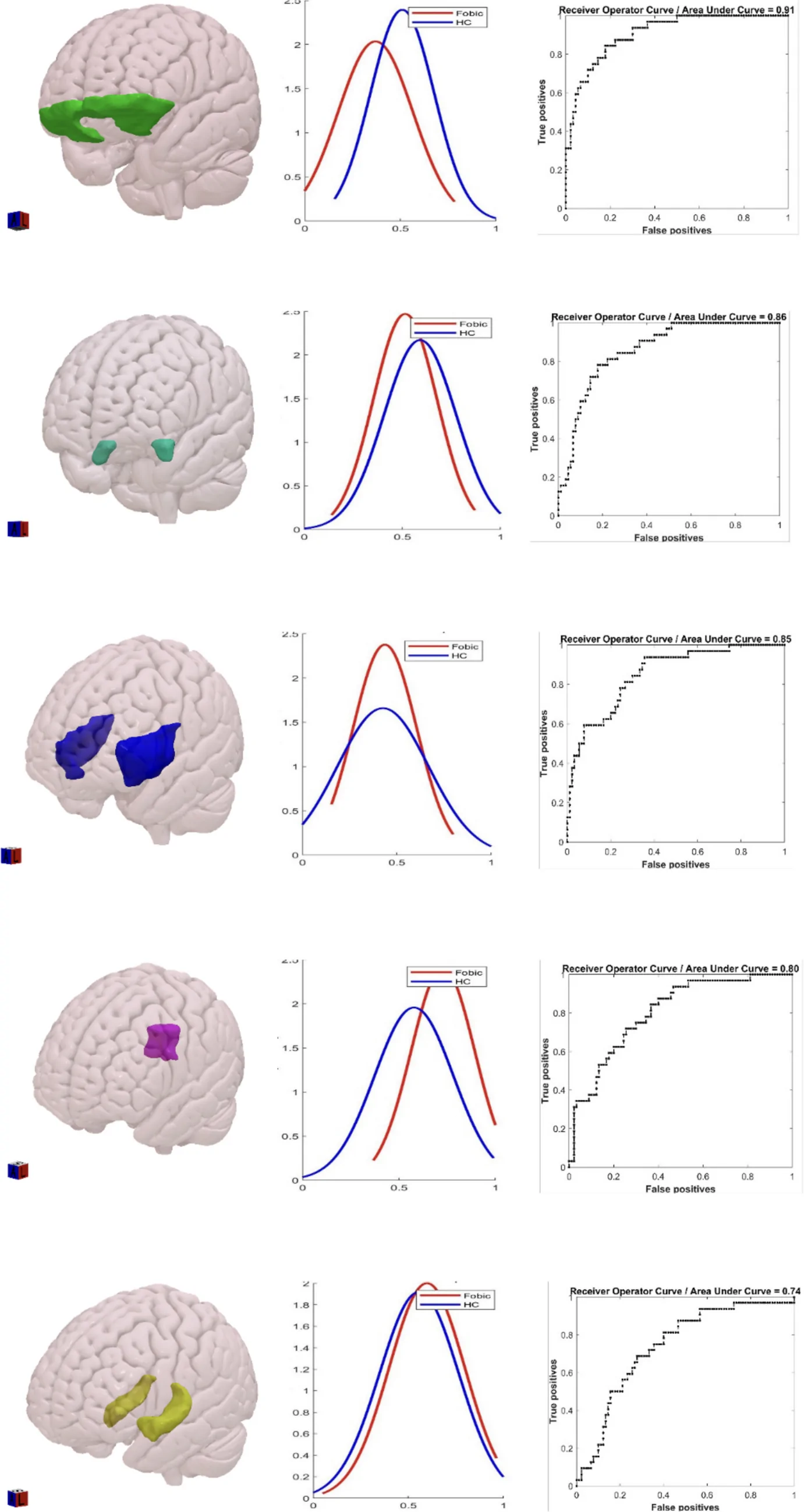
Neural results. Brain plots, histogram plots, and area under the curve plots of the predictive models ordered in terms of importance: the OFC, the amygdala, the insula, the posterior cingulate cortex, and the hippocampus**.** Of these regions, only the OFC, the amygdala, the insula, the posterior cingulate cortex exceeded the statistical threshold

**Fig. 3:**
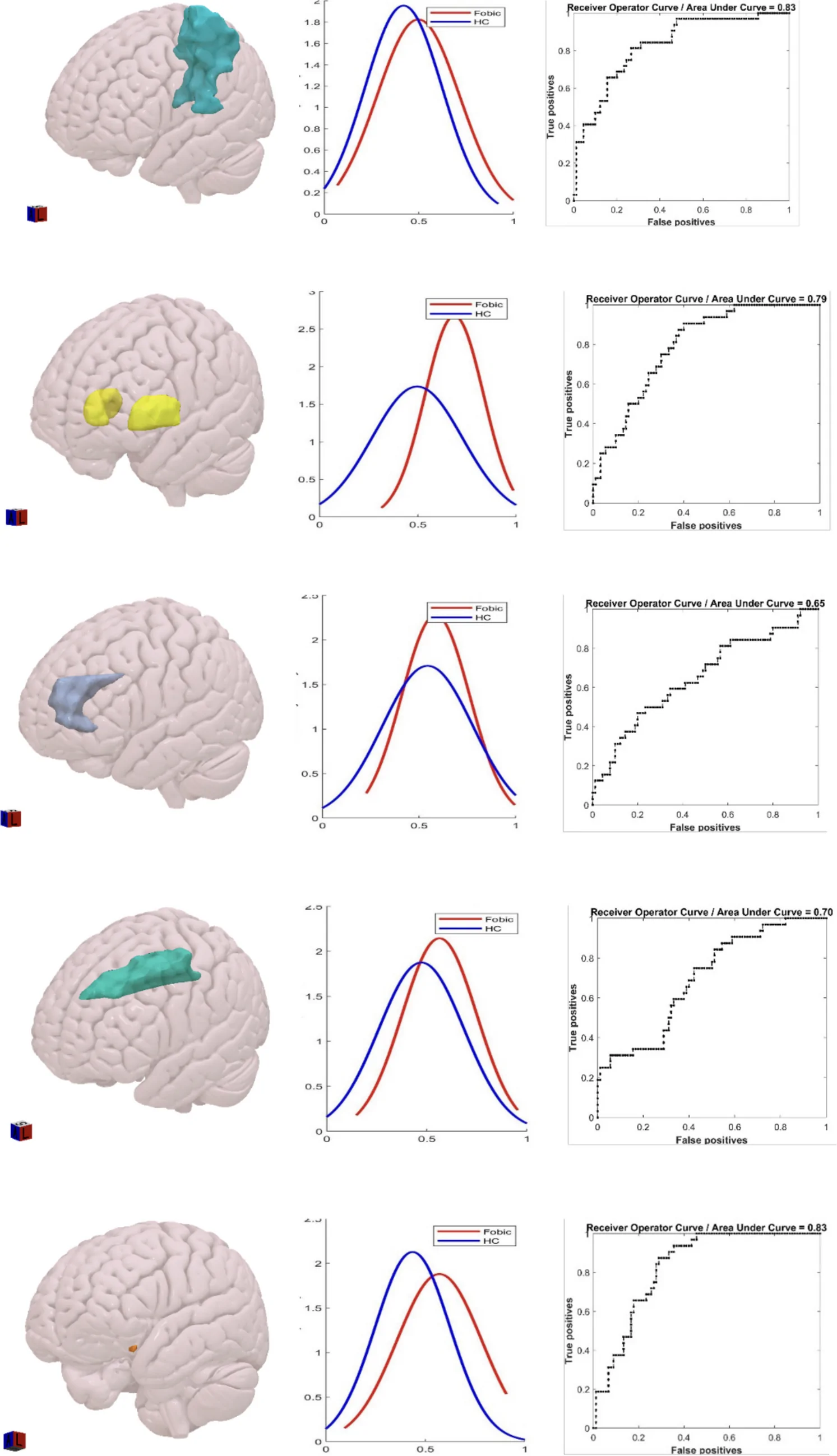
Neural results. Brain plots, histogram plots, and area under the curve plots of the predictive models ordered in terms of importance: the precuneus, the putamen, the anterior cingulate cortex, the middle cingulate cortex, and the hypothalamus. None of these regions exceeded the statistical threshold

However, only the orbitofrontal cortex and the amygdala displayed above chance (> 60%) values for all the parameters considered beside the BA (Precision, Sensitivity, Specificity, F1-Score). The other six brain areas examined did not consistently exceed chance levels on all parameters and not exceed the Bonferroni corrected threshold (Table 2; Figs. 2 and 3).

**Table 2:** Models’ evaluation

	Balanced accuracies	*p*	Area under the curve (AUC)	Precision	Sensitivity	Specificity	F1-Score
OFC	80.83%	0.0020*	0.91	0.67	0.75	0.87	0.71
Amygdala	76.15%	0.0020*	0.86	0.64	0.66	0.87	0.65
Insula	71.35%	0.0020*	0.85	0.56	0.59	0.83	0.58
Posterior cingulate cortex	71.25%	0.0020*	0.8	0.53	0.63	0.80	0.58
Hippocampus	66.11%	0.0040	0.74	0.5	0.5	0.82	0.5
Precuneus	65.87%	0.0100	0.83	0.62	0.41	0.91	0.49
Putamen	63.09%	0.0240	0.79	0.5	0.41	0.86	0.45
Anterior cingulate cortex	59.31%	0.0719	0.65	0.41	0.38	0.81	0.42
Middle cingulate cortex	56.08%	0.1417	0.7	0.35	0.34	0.78	0.34
Hypothalamus	50%	1.0000	-	-	-	-	-

Regions are displayed in order of importance according to the balanced accuracy. Please note that the additional parameters of the hypothalamus were not estimated due to its chance level BA performance

To further explore the relationship between brain structure and behavioral measures, we computed correlations between regional gray matter values and questionnaire scores. Overall, the results showed limited associations. Trend-level correlations (*p* < 0.08) were observed between the left amygdala and BAI scores (r = 0.325, *p* = 0.069), as well as between orbitofrontal cortex measures and SR scores (OFC left: r =  − 0.323, *p* = 0.071; OFC right: r =  − 0.32, *p* = 0.074). All remaining correlations were non-significant (*p* > 0.05).

## Discussion

The present study aimed to test the role of ten specific brain areas in classifying individuals with small animal phobia (SAP) from healthy controls. A supervised machine learning approach, known as Binary Support Vector Machine (BSVM), was applied to structural MRI images of SAP and healthy controls. We focused on ten brain structures previously associated with SAP: the amygdala, OFC, the insula, the anterior cingulate cortex, the middle cingulate cortex, the posterior cingulate cortex, the hippocampus, the precuneus, and the putamen. While previous studies have identified associations between these areas and SAP, a direct comparison of each region's specific contribution to SAP classification was lacking. Our results indicated that the OFC, amygdala, insula, and posterior cingulate cortex could correctly classify SAP individuals from controls. Of these regions, the OFC and amygdala displayed the best predictive performance across all statistical indices considered (all statistical indices above chance level). In the following sections, we discuss these findings in greater detail.

### The orbitofrontal cortex

Among the brain regions examined in the present study, the orbitofrontal cortex (OFC) demonstrated the highest classification accuracy in distinguishing individuals with SAP from healthy controls. The involvement of the OFC in anxiety disorders and specific phobias is well-documented (Del Casale et al., 2012; Hilbert et al., 2015; Rivero et al., 2023), so its strong predictive performance aligns with existing literature. Abnormalities in the OFC, particularly the left medial portion, have been linked to inappropriate fear reactions common in specific phobias (Hilbert et al., 2015). Functional MRI studies have shown increased OFC activation in individuals with dental phobia (Lueken et al., 2011) and alterations in various sections of the OFC across several anxiety disorders (Milad & Rauch, 2007). The OFC plays a crucial role in the extinction of previously learned fear responses—a process essential in fear conditioning and extinction learning (Milad & Rauch, 2007). Hypoactivation of the medial OFC (mOFC) may impair the inhibition of anxiety and fear responses, while hyperactivation of the anterolateral OFC has been associated with anxiety-related cognitions, such as rumination observed in obsessive–compulsive disorder (Milad & Rauch, 2007). Our findings underscore the complex role of the OFC in anxiety disorders, highlighting its importance not only in fear processing but also in the regulation of emotional responses. The high classification accuracy achieved by the OFC suggests it may serve as a key region for SAP, offering insights into targeted interventions focusing on OFC functioning to fear extinction.

### Amygdala

The amygdala emerged as the second most predictive region for SAP in our study, which is consistent with its well-established role in fear and anxiety processing. Previous research has highlighted the amygdala's involvement in specific phobias (Del Casale, 2012). Straube et al. (2006b) found increased activation in the right amygdala of individuals with spider phobia when exposed to phobic stimuli compared with neutral stimuli. Similarly, Larson et al. (2006) demonstrated that individuals with specific phobias exhibit shorter but more intense BOLD responses in the amygdala. Further studies have identified the amygdala as part of the "fear network," which includes regions such as the medial prefrontal cortex (mPFC), anterior cingulate cortex (ACC), insula, and thalamus, mediating fear responses in specific phobias (Schweckendiek et al., 2011). Our findings confirm that the amygdala carries sufficient information to accurately predict individuals with SAP, reinforcing its role as a central hub in fear processing and as a potential key region for anxiety disorders.

### Insula

The third most predictive region in our study was the insula. The insula plays a key role in mediating between environmental stimuli, autonomic system responses (such as physiological arousal), and higher-order cognitive processes (Wright et al., 2003). While often associated with mediating disgust reactions and empathy (Grecucci et al., 2013), the insula is also crucial in processing anxiety and fear responses.

The activation of the insula during the presentation of phobic stimuli is well-documented in the literature (Dilger et al., 2003; Straube et al., 2006a; b). Lueken et al. (2011) reported that insular activation varies with the phobia subtype, indicating its role in processing specific emotional and physiological responses. Their research found hyperactivation of the insula in individuals with snake phobia, while hypoactivation was observed in those with dental phobia (Lueken et al., 2011). The insula is also a key node (as well as the cingulate discussed later) in the Salience Network, known to be active when salient or dangerous stimuli are present in the environment (Baggio et al., 2023a, 2023b; Grecucci et al., 2023; Langerbeck et al., 2023). In our study, the insula proved to be a reliable classifier in distinguishing phobic individuals from healthy controls, supporting the findings sporadically reported in the literature.

### Cingulate cortex

The fourth and final region able to classify SAP from controls was the posterior cingulate cortex (PCC). We chose to analyze the cingulate cortex in three subsections due to confusion in the existing literature. In fact, the anterior (ACC) and posterior (PCC) portions of the cingulate cortex differ significantly in cytoarchitecture and are connected to distinct brain regions. Our results indicated that the PCC was predictive of SAP, while the anterior and middle parts of the cingulate cortex were not. There is substantial evidence in the literature supporting the role of the cingulate cortex in specific phobias. For example, Goossens et al. (2007) reported increased bilateral activation of the ACC in an fMRI experiment when phobic subjects were exposed to images related to their phobia. This is consistent with the idea that the cingulate cortex is involved in processing potentially threatening or emotionally significant stimuli (Bush et al., 2000). Similarly, Straube et al. (2006b) observed cingulate activation during a task where phobic subjects were shown images of spiders (phobic stimulus) and mushrooms (neutral stimulus), reinforcing the ACC’s role in processing threatening stimuli in phobics. In contrast, the role of the PCC in specific phobias has received less empirical support. However, Straube et al. (2006a) reported enhanced PCC activity in phobic individuals during the presentation of phobic stimuli in a linguistic form. The authors suggested that this activation might reflect increased mnemonic processing related to learning about phobic stimuli in individuals with specific phobias.

All other regions studied, namely the middle cingulate cortex, the hippocampus, the precuneus, and the putamen, did not survive the Bonferroni corrected threshold. Previous studies have reported some of these regions to be involved in phobias. Based on our results we cannot exclude that these regions may play a role in phobias, but in the comparison with the other structures they certainly have less predictive power.

### Limitations, future directions, and translational possibilities

The present study demonstrates the potential of using Machine Learning methods to examine the role of specific brain regions in classifying individuals with small animal phobia (SAP). These findings could eventually lead to the development of predictive models or biomarkers capable of identifying significant brain alterations in specific anxiety disorders. However, despite its merits, the study has several limitations that should be acknowledged. The first limitation lies in the exclusive use of gray matter structural data. Future research should incorporate white matter analyses and functional data to build more comprehensive models of the complex neurophysiological interactions underlying specific phobias. Second, the relatively small sample size, while larger than many previous studies, still restricts the generalizability of the results. Expanding the sample size, particularly regarding the number of phobic subjects, is crucial for improving the robustness and applicability of the findings. Third, we conducted bilateral analyses of brain regions without exploring potential hemispheric lateralization. According to the literature, many of these regions show functional lateralization of activation in the context of specific phobias. It would be valuable to explore whether significant structural differences, such as in gray matter volume, are also lateralized. Fourth, focusing solely on individual brain regions might not fully capture the complex neural interactions involved in SAP. In a previous study (Scarano et al., 2025), a network-based approach was used and reported that some large-scale brain networks, such as the Default Mode Network and the Affective Network, significantly contributed to predict SAP. However, these networks included many regions and the exact contribution of each region was not assessed. Given the structural nature of the data, these findings should be interpreted in terms of association and discriminative information rather than direct functional encoding or causal mechanisms. Importantly, while the present study focused on the classification performance of predefined regions, the underlying SVM models are inherently interpretable at the voxel level through weight maps. These maps can provide qualitative indications of which subregions within each ROI contribute most to the classification. However, such weights should be interpreted with caution, as they reflect multivariate decision boundaries rather than direct neurobiological relevance (Haufe et al., 2014). Future studies could extend the present approach by explicitly characterizing subregional patterns within each structure. Of note, the regions identified in the present study, including the orbitofrontal cortex, amygdala, insula, and cingulate cortex, are consistently implicated across a wide range of anxiety and stress-related conditions. Therefore, the present findings should be interpreted cautiously, because they may reflect transdiagnostic mechanisms of fear and anxiety rather than a disorder-specific neural signature of small animal phobia. In the absence of comparison groups with other anxiety disorders and dimensional analyses of anxiety traits across the full sample, the specificity of these patterns to SAP cannot be firmly established. Finally, future studies should consider exploring differences between various types of specific phobias.

Our findings suggest the main role of both OFC and amygdala brain regions to differentiate between phobic and non-phobic individuals. These results are in line with those that link determinate brain activation changes as neural explanation why psychological treatment works. Specifically, there is a model emphasizing the role of two main brain areas in the efficacy of psychological therapies on anxiety disorders: an increased activation of brain areas associated with (adequate) emotion regulation, such as OFC, and a (consequent) decrement activation in limbic areas, such as the amygdala (Lueken & Hahn, 2016). Even though there is some controversy in verifying this dual model (Viña et al., 2020), there is a steady support observed in several research data (Marwood et al., 2018).

Additionally, these areas can be the target of possible neurostimulation interventions via transcranial direct electrical stimulation (tDCS) or transcranial magnetic stimulation (TMS) to help individuals with SAP to reduce their symptoms, and in combination with psychological therapies to facilitate change and reduce the time of intervention.

## Conclusion

This study's findings may open up new research directions aimed at better understanding the neural basis of SAP. Once these mechanisms are clearly identified, neurostimulation interventions targeting specific brain regions could be developed to alleviate the symptoms experienced by individuals with SAP.

## Data Availability

Data will be made available upon reasonable request.

## References

[CR1] Ahmadi Ghomroudi, P., Siugzdaite, R., Messina, I., & Grecucci, A. (2024). Decoding acceptance and reappraisal strategies from resting state macro networks. *Scientific Reports,**14*, Article 19232. 10.1038/s41598-024-68490-9 PMC11336109 39164353

[CR2] Baggio, T., Grecucci, A., Crivello, F., Marc, J., & Tzourio, C. (2023a). Fronto-parietal and cerebellar circuits characterize individuals with higher trait anxiety. *European Journal of Neuroscience,**62*(3), Article e70210. 10.1111/ejn.70210 40758310

[CR3] Baggio, T., Grecucci, A., Meconi, F., & Messina, I. (2023b). Anxious brains: A combined data fusion machine learning approach to predict trait anxiety from morphometric features. *Sensors*. 10.3390/s23020610 PMC9863274 36679404

[CR4] Barrett, L. F., & Satpute, A. B. (2013). Large-scale brain networks in affective and social neuroscience: Towards an integrative functional architecture of the brain. *Current Opinion in Neurobiology,**23*(3), 361–372. 10.1016/j.conb.2012.12.012 PMC4119963 23352202

[CR5] Bechara, A., Damasio, H., Damasio, A. R., & Lee, G. P. (1999). Different contributions of the human amygdala and ventromedial prefrontal cortex to decision-making. *The Journal of Neuroscience,**19*(13), 5473–5481. 10.1523/JNEUROSCI.19-13-05473.1999 PMC6782338 10377356

[CR6] Bechara, A., Damasio, H., Tranel, D., & Damasio, A. R. (1997). Deciding advantageously before knowing the advantageous strategy. *Science,**275*(5304), 1293–1295. 10.1126/science.275.5304.1293 9036851

[CR7] Beck, A. T., Epstein, N., Brown, G., & Steer, R. A. (1988). An inventory for measuring clinical anxiety: Psychometric properties. *Journal of Consulting and Clinical Psychology,**56*(6), 893–897. 10.1037/0022-006X.56.6.893 3204199

[CR8] Böhnlein, J., Leehr, E. J., Roesmann, K., Sappelt, T., Platte, O., Grotegerd, D., Sindermann, L., Repple, J., Opel, N., Meinert, S., Lemke, H., Borgers, T., Dohm, K., Enneking, V., Goltermann, J., Waltemate, L., Hülsmann, C., Thiel, K., Winter, N. R., … Dannlowski, U. (2021). Neural processing of emotional facial stimuli in specific phobia: An fMRI study. *Depression and Anxiety,**38*(8), 846–859. 10.1002/da.23191 34224655

[CR9] Buckner, R. L., Andrews-Hanna, J. R., & Schacter, D. L. (2008). The brain’s default network: Anatomy, function, and relevance to disease. *Annals of the New York Academy of Sciences,**1124*, 1–38. 10.1196/annals.1440.011 18400922

[CR10] Bush, G., Luu, P., & Posner, M. I. (2000). Cognitive and emotional influences in anterior cingulate cortex. *Trends in Cognitive Sciences,**4*(6), 215–222. 10.1016/s1364-6613(00)01483-2 10827444

[CR11] Calhoun, V. D., Liu, J., & Adali, T. (2009). A review of group ICA for fMRI data and ICA for joint inference of imaging, genetic, and ERP data. *NeuroImage,**45*(1 Suppl), S163-172. 10.1016/j.neuroimage.2008.10.057 PMC2651152 19059344

[CR12] Claesen, M., & De Moor, B. (2015). *Hyperparameter search in machine learning* (arXiv: 1502.02127). *arXiv*. 10.48550/arXiv.1502.02127

[CR13] Craske, M. G., Rauch, S. L., Ursano, R., Prenoveau, J., Pine, D. S., & Zinbarg, R. E. (2011). What is an anxiety disorder? *Focus,**9*(3), 369–388. 10.1176/foc.9.3.foc369 19957279

[CR14] Damoiseaux, J. S., Rombouts, S. A., Barkhof, F., Scheltens, P., Stam, C. J., Smith, S. M., & Beckmann, C. F. (2006). Consistent resting-state networks across healthy subjects. *Proceedings of the National Academy of Sciences of the United States of America,**103*(37), Article Article 1. 10.1073/pnas.0601417103 PMC1564249 16945915

[CR15] Del Casale, A., Ferracuti, S., Rapinesi, C., Serata, D., Piccirilli, M., Savoja, V., Kotzalidis, G. D., Manfredi, G., Angeletti, G., Tatarelli, R., & Girardi, P. (2012). Functional neuroimaging in specific phobia. *Psychiatry Research: Neuroimaging,**202*(3), 181–197. 10.1016/j.pscychresns.2011.10.009 22804970

[CR16] Dilger, S., Straube, T., Mentzel, H. J., Fitzek, C., Reichenbach, J. R., Hecht, H., Krieschel, S., Gutberlet, I., & Miltner, W. H. (2003). Brain activation to phobia-related pictures in spider phobic humans: An event-related functional magnetic resonance imaging study. *Neuroscience Letters,**348*(1), 29–32. 10.1016/s0304-3940(03)00647-5 12893418

[CR17] Doll, A., Sorg, C., Manoliu, A., Woller, A., Meng, C., Forstl, H., Zimmer, C., Wohlschlager, A. M., & Riedl, V. (2013). Shifted intrinsic connectivity of central executive and salience network in borderline personality disorder. *Frontiers in Human Neuroscience*, *7*. 10.3389/fnhum.2013.00727. PMC3812906 24198777

[CR18] Dörfel, D., Gärtner, A., & Scheffel, C. (2020). Resting state cortico-limbic functional connectivity and dispositional use of emotion regulation strategies: A replication and extension study. *Frontiers in Behavioral Neuroscience,**14*, 128–128. 10.3389/fnbeh.2020.00128 PMC7399345 32848654

[CR19] Doucet, G. E., Lee, W. H., & Frangou, S. (2019). Evaluation of the spatial variability in the major resting-state networks across human brain functional atlases. *Human Brain Mapping,**40*(15), 4577–4587. 10.1002/hbm.24722 PMC6771873 31322303

[CR20] Enatsu, R., Gonzalez-Martinez, J., Bulacio, J., Kubota, Y., Mosher, J., Burgess, R. C., Najm, I., & Nair, D. R. (2015). Connections of the limbic network: A corticocortical evoked potentials study. *Cortex,**62*, 20–33. 10.1016/j.cortex.2014.06.018 25131616

[CR21] Endler, N. S., Hunt, J. M. C. V., & Rosenstein, A. J. (1962). An SR inventory of anxiousness. *Psychological Monographs: General and Applied,**76*(17), 1.

[CR22] Etkin, A., & Wager, T. D. (2007). Functional neuroimaging of anxiety: A meta-analysis of emotional processing in PTSD, social anxiety disorder, and specific phobia. *The American Journal of Psychiatry,**164*(10), 1476–1488. 10.1176/appi.ajp.2007.07030504 PMC3318959 17898336

[CR23] Fisler, M. S., Federspiel, A., Horn, H., Dierks, T., Schmitt, W., Wiest, R., de Quervain, D.-F., & Soravia, L. M. (2013). Spider phobia is associated with decreased left amygdala volume: A cross-sectional study. *BMC Psychiatry,**13*(1), Article 70. 10.1186/1471-244X-13-70 PMC3599010 23442196

[CR24] Fortin, J.-P., Cullen, N., Sheline, Y. I., Taylor, W. D., Aselcioglu, I., Cook, P. A., Adams, P., Cooper, C., Fava, M., McGrath, P. J., McInnis, M., Phillips, M. L., Trivedi, M. H., Weissman, M. M., & Shinohara, R. T. (2018). Harmonization of cortical thickness measurements across scanners and sites. *NeuroImage,**167*, 104–120. 10.1016/j.neuroimage.2017.11.024 PMC5845848 29155184

[CR25] Frick, A., Gingnell, M., Marquand, A. F., Howner, K., Fischer, H., & Kristiansson, M. (2014). *Classifying social anxiety disorder using multivoxel pattern analyses of brain function and structure* (Vol. 259, pp. 330–335). 10.1016/j.bbr.2013.11.003 PMC3888925 24239689

[CR26] Fyer, A. J. (1998). Current approaches to etiology and pathophysiology of specific phobia. *Biological Psychiatry,**44*(12), 1295–1304. 10.1016/S0006-3223(98)00274-1 9861472

[CR27] Gentili, C., Messerotti Benvenuti, S., Lettieri, G., Costa, C., & Cecchetti, L. (2019). ROI and phobias: The effect of ROI approach on an ALE meta-analysis of specific phobias. *Human Brain Mapping,**40*(6), 1814–1828. 10.1002/hbm.24492 PMC6865604 30548734

[CR28] Gorgolewski, K. J., Durnez, J., & Poldrack, R. A. (2017). Preprocessed consortium for neuropsychiatric phenomics dataset. *F1000Research,**6*, Article 1262. 10.12688/f1000research.11964.2 PMC5664981 29152222

[CR29] Grahn, J. A., Parkinson, J. A., & Owen, A. M. (2008). The cognitive functions of the caudate nucleus. *Progress in Neurobiology,**86*(3), 141–155. 10.1016/j.pneurobio.2008.09.004 18824075

[CR30] Grecucci, A., Dadomo, H., Salvato, G., Lapomarda, G., Sorella, S., & Messina, I. (2023). *Abnormal Brain Circuits Characterize Borderline Personality and Mediate the Relationship between Childhood Traumas and Symptoms: A mCCA+jICA and*. 10.3390/s23052862 PMC10006907 36905064

[CR31] Grecucci, A., Giorgetta, C., Van’t Wout, M., Bonini, N., & Sanfey, A. G. (2013). Reappraising the ultimatum: An fMRI study of emotion regulation and decision making. *Cerebral Cortex,**23*(2), 399–410. 10.1093/cercor/bhs028 22368088

[CR32] Grecucci, A., Lapomarda, G., Messina, I., Monachesi, B., Sorella, S., & Siugzdaite, R. (2022). Structural features related to affective instability correctly classify patients with borderline personality disorder. A supervised machine learning approach. *Frontiers in Psychiatry*. 10.3389/fpsyt.2022.804440 PMC8918568 35295769

[CR33] Grecucci, A., Rubicondo, D., Siugzdaite, R., Surian, L., & Job, R. (2016). Uncovering the social deficits in the autistic brain. A source-based morphometric study. *Frontiers in Neuroscience,**10*, 388. 10.3389/fnins.2016.00388 PMC5005369 27630538

[CR34] Grecucci, A., Scarano, A., Fumero, A., Rivero, F., Marrero, R. J., Olivares, T., Álvarez-Pérez, Y., & Peñate, W. (2025). The two sides of Phobos: Gray and white matter abnormalities in phobic individuals. *Cognitive & Affective Behavioral Neuroscience,**25*(2), 550–569. 10.3758/s13415-024-01258-w 39753800

[CR35] Griffanti, L., Douaud, G., Bijsterbosch, J., Evangelisti, S., Alfaro-Almagro, F., Glasser, M. F., Duff, E. P., Fitzgibbon, S., Westphal, R., Carone, D., Beckmann, C. F., & Smith, S. M. (2017). Hand classification of fMRI ICA noise components. *NeuroImage,**154*, 188–205. 10.1016/j.neuroimage.2016.12.036 PMC5489418 27989777

[CR36] Goossens, P. J. J., Van Achterberg, T. & Knoppert-van der Klein, E. A. M. (2007). Nursing processes used in the treatment of patients with bipolar disorder. *International Journal of Mental Health Nursing*, 16:168–177. 10.1111/j.1447-0349.2007.00464.x 17535162

[CR37] Haber, S. N., & Knutson, B. (2010). The reward circuit: Linking primate anatomy and human imaging. *Neuropsychopharmacology,**35*(1), 4–26. 10.1038/npp.2009.129 PMC3055449 19812543

[CR38] Hamilton, M. (1959). The assessment of anxiety states by rating. *British Journal of Medical Psychology*. 10.1111/j.2044-8341.1959.tb00467.x 13638508

[CR39] Hastie, T., R. Tibshirani, and J. Friedman. *The elements of statistical learning*, 2nd edition. Springer; 2009

[CR40] Haufe, S., Meinecke, F., Görgen, K., Dähne, S., Haynes, J.-D., Blankertz, B., & Bießmann, F. (2014). On the interpretation of weight vectors of linear models in multivariate neuroimaging. *NeuroImage,**87*, 96–110. 10.1016/j.neuroimage.2013.10.067 24239590

[CR41] Hilbert, K., Evens, R., Maslowski, N. I., Wittchen, H. U., & Lueken, U. (2015). Neurostructural correlates of two subtypes of specific phobia: A voxel-based morphometry study. *Psychiatry Research Neuroimaging,**231*, 168–175. 10.1016/j.pscychresns.2014.12.003 25561374

[CR42] Hilbert, K., Lueken, U., & Beesdo-Baum, K. (2014). Neural structures, functioning and connectivity in generalized anxiety disorder and interaction with neuroendocrine systems: A systematic review. *Journal of Affective Disorders,**158*, 114–126. 10.1016/j.jad.2014.01.022 24655775

[CR43] Hofmann, T., Scholkopf, B., & Smola, A. J. (2008). Kernel methods in machine learning. *Annals of Statistics,**36*(3), 1171–1220.

[CR44] Janes, A. C., Nickerson, L. D., Frederick, B., & Kaufman, M. J. (2012). Prefrontal and limbic resting state brain network functional connectivity differs between nicotine-dependent smokers and non-smoking controls. *Drug and Alcohol Dependence,**125*(3), 252–259. 10.1016/j.drugalcdep.2012.02.020 PMC3389311 22459914

[CR45] Jornkokgoud, K., Baggio, T., Bakiaj, R., Wongupparaj, P., Job, R., & Grecucci, A. (2024). Narcissus reflected: Grey and white matter features joint contribution to the default mode network in predicting narcissistic personality traits. *European Journal of Neuroscience,**59*(12), 3273–3291. 10.1111/ejn.16345 38649337

[CR46] Jornkokgoud, K., Baggio, T., Faysal, M., Bakiaj, R., Wongupparaj, P., Job, R., & Grecucci, A. (2023). Predicting narcissistic personality traits from brain and psychological features: A supervised machine learning approach. *Social Neuroscience,**18*(5), 257–270. 10.1080/17470919.2023.2242094 37497589

[CR47] Kendler, K. S., Myers, J., Prescott, C. A., & Neale, M. C. (2001). The genetic epidemiology of irrational fears and phobias in men. *Archives of General Psychiatry,**58*(3), 257–265. 10.1001/archpsyc.58.3.257 11231833

[CR48] Kessler, R. C., & Üstün, T. B. (2004). The World Mental Health (WMH) survey initiative version of the World Health Organization (WHO) composite international diagnostic interview (CIDI. *International Journal of Methods in Psychiatric Research,**13*, 93–121. 10.1002/mpr.168 PMC6878592 15297906

[CR49] Langerbeck, M., Baggio, T., Messina, I., Bhat, S., & Grecucci, A. (2023). Borderline shades: Morphometric features predict borderline personality traits but not histrionic traits. *NeuroImage: Clinical,**40*, Article 103530. 10.1016/j.nicl.2023.103530 PMC10618757 37879232

[CR50] Larson, C. L., Schaefer, H. S., Siegle, G. J., Jackson, C. A., Anderle, M. J., & Davidson, R. J. (2006). Fear is fast in phobic individuals: Amygdala activation in response to fear-relevant stimuli. *Biological Psychiatry,**60*(4), 410–417. 10.1016/j.biopsych.2006.03.079 16919528

[CR51] LeDoux, J. E. (2000). Emotion circuits in the brain. *Annual Review of Neuroscience,**23*, 155–184. 10.1146/annurev.neuro.23.1.155 10845062

[CR52] Lueken, U., & Hahn, T. (2016). Functional neuroimaging of psychotherapeutic processes in anxiety and depression: From mechanisms to predictions. *Current Opinion in Psychiatry,**29*, 25–31. 10.1097/YCO.0000000000000218 26651007

[CR53] Lueken, U., Hilbert, K., Wittchen, H.-U., Reif, A., & Hahn, T. (2015). Diagnostic classification of specific phobia subtypes using structural MRI data: A machine-learning approach. *Journal of Neural Transmission,**122*(1), 123–134. 10.1007/s00702-014-1272-5 25037587

[CR54] Lueken, U., Kruschwitz, J. D., Muehlhan, M., Siegert, J., Hoyer, J., & Wittchen, H. U. (2011) How specific is specific phobia? Different neural response patterns in two subtypes of specific phobia. *Neuroimage*, *56*(1):363–72. 10.1016/j.neuroimage.2011.02.015 21316468

[CR55] Luo, N., Sui, J., Abrol, A., Chen, J., Turner, J. A., Damaraju, E., Fu, Z., Fan, L., Lin, D., Zhuo, C., Xu, Y., Glahn, D. C., Rodrigue, A. L., Banich, M. T., Pearlson, G. D., & Calhoun, V. D. (2020). Structural brain architectures match intrinsic functional networks and vary across domains: A study from 15 000+ individuals. *Cerebral Cortex,**30*(10), 5460–5470. 10.1093/cercor/bhaa127 PMC7566687 32488253

[CR56] Mah, L., Szabuniewicz, C., & Fiocco, A. J. (2016). Can anxiety damage the brain? *Current Opinion in Psychiatry,**29*, 56–63. 10.1097/YCO.0000000000000223 26651008

[CR57] Marwood, L., Wise, T., Perkins, A. M., & Cleare, A. J. (2018). Meta-analyses of the neural mechanisms and predictors of response to psychotherapy in depression and anxiety. *Neuroscience and Biobehavioral Reviews,**95*, 61–72. 10.1016/j.neubiorev.2018.09.022 PMC6267850 30278195

[CR58] McKeown, M. J., Makeig, S., Brown, G. G., Jung, T.-P., Kindermann, S. S., Bell, A. J., & Sejnowski, T. J. (1998). Analysis of fMRI data by blind separation into independent spatial components. *Human Brain Mapping,**6*, 160–188. 10.1002/(SICI)1097-0193(1998)6:3<160::AID-HBM5>3.0.CO;2-1 PMC6873377 9673671

[CR59] Meier, J., Tewarie, P., Hillebrand, A., Douw, L., van Dijk, B. W., Stufflebeam, S. M., & Van Mieghem, P. (2016). A mapping between structural and functional brain networks. *Brain Connectivity,**6*(4), 298–311. 10.1089/brain.2015.0408 PMC4939447 26860437

[CR60] Milad, M. R., & Rauch, S. L. (2007). The role of the orbitofrontal cortex in anxiety disorders. *Annals of the New York Academy of Sciences,**1121*, 546–561. 10.1196/annals.1401.006 17698998

[CR61] Monté-Rubio, G. C., Falcón, C., Pomarol-Clotet, E., & Ashburner, J. (2018). A comparison of various MRI feature types for characterizing whole brain anatomical differences using linear pattern recognition methods. *NeuroImage,**178*, 753–768. 10.1016/j.neuroimage.2018.05.065 PMC6202442 29864520

[CR62] Mourao-Miranda, J., Almeida, J., Hassel, S., Oliveira, L., Versace, A., & Marquand, A. (2012). Pattern recognition analyses of brain activation elicited by happy and neutral faces in unipolar and bipolar depression. *Bipolar Disorders,**14*, 451–460. 10.1111/j.1399-5618.2012.01019.x PMC3510302 22631624

[CR63] Murphy, F. C., Nimmo-Smith, I., & Lawrence, A. D. (2003). Functional neuroanatomy of emotions: A meta-analysis. *Cognitive, Affective & Behavioral Neuroscience,**3*(3), 207–233. 10.3758/cabn.3.3.207 14672157

[CR64] Mwangi, B., Ebmeier, K. P., Matthews, K., & Steele, J. D. (2012). Multi-centre diagnostic classification of individual structural neuroimaging scans from patients with major depressive disorder. *Brain,**135*, 1508–1521. 10.1093/brain/aws084 22544901

[CR65] Norman, K. A., Polyn, S. M., Detre, G. J., & Haxby, J. V. (2006). Beyond mind-reading: Multivoxel pattern analysis of fMRI data. *Trends in Cognitive Sciences,**10*(9), 424–430. 10.1016/j.tics.2006.07.005 16899397

[CR66] Olatunji, B. O. (Ed.). (2019). *The Cambridge handbook of anxiety and related disorders*. Cambridge University Press. 10.1017/9781108140416

[CR67] Olson, I. R., Plotzker, A., & Ezzyat, Y. (2007). The enigmatic temporal pole: A review of findings on social and emotional processing. *Brain,**130*(Pt 7), 1718–1731. 10.1093/brain/awm052 17392317

[CR68] Packard, M. G., & Goodman, J. (2013). Factors that influence the relative use of multiple memory systems. *Hippocampus,**23*(11), 1044–1052. 10.1002/hipo.22178 23929809

[CR69] Pappaianni, E., Pisapia, N., Siugzdaite, R., Crescentini, C., A., C., & Job, R. (2019). Less is more: Psychological and morphometric differences between low vs high reappraisers. *Cognitive & Affective Behavioral Neuroscience*. 10.3758/s13415-019-00757-5 PMC7613187 31858436

[CR70] Pappaianni, E., Siugzdaite, R., Vettori, S., Venuti, P., Job, R., & Grecucci, A. (2018). Three shades of grey: Detecting brain abnormalities in children with autism using source-, voxel- and surface-based morphometry. *European Journal of Neuroscience,**47*, Article 13704. 10.1111/ejn.13704 28921735

[CR71] Paulus, M. P., & Stein, M. B. (2006). An insular view of anxiety. *Biological Psychiatry,**60*(4), 383–387. 10.1016/j.biopsych.2006.03.042 16780813

[CR72] Peñate, W., Fumero, A., Viña, C., Herrero, M., Marrero, R. J., & Rivero, F. (2017). A meta-analytic review of neuroimaging studies of specific phobia to small animals. *The European Journal of Psychiatry,**31*(1), 23–36. 10.1016/j.ejpsy.2016.12.003

[CR73] Phelps, E. A., Delgado, M. R., Nearing, K. I., & LeDoux, J. E. (2004). Extinction learning in humans: Role of the amygdala and vmPFC. *Neuron,**43*(6), 897–905. 10.1016/j.neuron.2004.08.042 15363399

[CR74] Pisner, A., Schnyer, D.M. (2020). Support vector machine. Machine Learning, Elsevier, 2020, pp. 101–121

[CR75] Pruim, R. H. R., Mennes, M., Buitelaar, J. K., & Beckmann, C. F. (2015). ICA-AROMA: A robust ICA-based strategy for removing motion artifacts from fMRI data. *NeuroImage,**112*, 267–277. 10.1016/j.neuroimage.2015.02.064 25770991

[CR76] Raichle, M. E., MacLeod, A. M., Snyder, A. Z., Powers, W. J., Gusnard, D. A., & Shulman, G. L. (2001). A default mode of brain function. *Proceedings of the National Academy of Sciences,**98*(2), 676–682. 10.1073/pnas.98.2.676 PMC14647 11209064

[CR77] Rauch, S. L., Shin, L. M., & Wright, C. I. (2003). Neuroimaging studies of amygdala function in anxiety disorders. *Annals of the New York Academy of Sciences,**985*, 389–410. 10.1111/j.1749-6632.2003.tb07096.x 12724173

[CR78] Rauch, S. L., Wright, C. I., Martis, B., Busa, E., McMullin, K. G., Shin, L. M., Dale, A. M., & Fischl, B. (2004). A magnetic resonance imaging study of cortical thickness in animal phobia. *Biological Psychiatry,**55*(9), 946–952. 10.1016/j.biopsych.2003.12.022 15110739

[CR79] Rivero, F., Marrero, R. J., Olivares, T., Peñate, W., Álvarez-Pérez, Y., Bethencourt, J. M., & Fumero, A. (2023). A voxel-based morphometric study of gray matter in specific phobia. *Life,**13*(1), 1. 10.3390/life13010119 PMC9864817 36676068

[CR80] Rondina, J. M., Ferreira, L. K., Souza Duran, F. L., Kubo, R., Ono, C. R., Leite, C. C., Smid, J., Nitrini, R., Buchpiguel, C. A., & Busatto, G. F. (2018). Selecting the most relevant brain regions to discriminate Alzheimer’s disease patients from healthy controls using multiple kernel learning: A comparison across functional and structural imaging modalities and atlases. *NeuroImage: Clinical,**17*, 628–641. 10.1016/j.nicl.2017.10.026 PMC5716956 29234599

[CR81] Rosso, I. M., Makris, N., Britton, J. C., Price, L. M., Gold, A. L., Zai, D., Bruyere, J., Deckersbach, T., Killgore, W. D. S., & Rauch, S. L. (2010). Anxiety sensitivity correlates with two indices of right anterior insula structure in specific animal phobia. *Depression and Anxiety,**27*(12), 1104–1110. 10.1002/da.20765 PMC3010373 21132846

[CR82] Santomauro, D. F., Mantilla Herrera, A. M., Shadid, J., Zheng, P., Ashbaugh, C., Pigott, D. M., Abbafati, C., Adolph, C., Amlag, J. O., Aravkin, A. Y., Bang-Jensen, B. L., Bertolacci, G. J., Bloom, S. S., Castellano, R., Castro, E., Chakrabarti, S., Chattopadhyay, J., Cogen, R. M., Collins, J. K., … Ferrari, A. J. (2021). Global prevalence and burden of depressive and anxiety disorders in 204 countries and territories in 2020 due to the COVID-19 pandemic. *The Lancet,**398*(10312), 1700–1712. 10.1016/S0140-6736(21)02143-7 PMC8500697 34634250

[CR83] Scarano, A., Fumero, A., Baggio, T., Rivero, F., Marrero, R. J., Olivares, T., Peñate, W., Álvarez-Pérez, Y., Bethencourt, J. M., & Grecucci, A. (2025). The phobic brain: Morphometric features correctly classify individuals with small animal phobia. *Psychophysiology,**62*(1), Article e14716. 10.1111/psyp.14716 PMC11785541 39467845

[CR84] Schweckendiek, J., Klucken, T., Merz, C. J., Tabbert, K., Walter, B., Ambach, W., Vaitl, D., & Stark, R. (2011). Weaving the (neuronal) web: Fear learning in spider phobia. *Neuroimage*, *54*(1):681–8. 10.1016/j.neuroimage.2010.07.049 20673801

[CR85] Schienle, A., Ebner, F., & Schäfer, A. (2011). Localized gray matter volume abnormalities in generalized anxiety disorder. *European Archives of Psychiatry and Clinical Neuroscience,**261*(4), 303–307. 10.1007/s00406-010-0147-5 20820793

[CR86] Schmahmann, J. D. (2019). The cerebellum and cognition. *Neuroscience Letters,**688*, 62–75. 10.1016/j.neulet.2018.07.005 29997061

[CR87] Schrouff, J., Rosa, M. J., Rondina, J. M., Marquand, A. F., Chu, C., Ashburner, J., Phillips, C., Richiardi, J., & Mourão-Miranda, J. (2013). PRoNTo: Pattern recognition for neuroimaging toolbox. *Neuroinformatics,**11*(3), 319–337. 10.1007/s12021-013-9178-1 PMC3722452 23417655

[CR88] Schutter, D. J. L. G., & van Honk, J. (2005). The cerebellum on the rise in human emotion. *Cerebellum (London, England),**4*(4), 290–294. 10.1080/14734220500348584 16321885

[CR89] Seeley, W. W., Menon, V., Schatzberg, A. F., Keller, J., Glover, G. H., Kenna, H., Reiss, A. L., & Greicius, M. D. (2007). Dissociable intrinsic connectivity networks for salience processing and executive control. *The Journal of Neuroscience,**27*(9), 2349–2356. 10.1523/JNEUROSCI.5587-06.2007 PMC2680293 17329432

[CR90] Sherman, S. M. (2007). The thalamus is more than just a relay. *Current Opinion in Neurobiology,**17*(4), 417–422. 10.1016/j.conb.2007.07.003 PMC2753250 17707635

[CR91] Shin, L. M., & Liberzon, I. (2010). The neurocircuitry of fear, stress, and anxiety disorders. *Neuropsychopharmacology,**35*(1), 169–191. 10.1038/npp.2009.83 PMC3055419 19625997

[CR92] Sorella, S., Lapomarda, G., Messina, I., Frederickson, J. J., Siugzdaite, R., & Job, R. (2019). Testing the expanded continuum hypothesis of schizophrenia and bipolar disorder. Neural and psychological evidence for shared and distinct mechanisms. *NeuroImage: Clinical,**23*, Article 101854. 10.1016/j.nicl.2019.101854 PMC6529770 31121524

[CR93] Squarcina, L., Bellani, M., Rossetti, M. G., Perlini, C., Delvecchio, G., & Dusi, N. (2017). Similar white matter changes in schizophrenia and bipolar disorder: A tract-based spatial statistics study. *PLoS ONE,**12*, Article 0178089. 10.1371/journal.pone.0178089 PMC5489157 28658249

[CR94] Squire, L. R., Stark, C. E. L., & Clark, R. E. (2004). The medial temporal lobe. *Annual Review of Neuroscience,**27*, 279–306. 10.1146/annurev.neuro.27.070203.144130 15217334

[CR95] Straube, T., Glauer, M., Dilger, S., Mentzel, H. J., & Miltner, W. H. (2006a) Effects of cognitive-behavioral therapy on brain activation in specific phobia. *Neuroimage*, *29*(1):125–35. 10.1016/j.neuroimage.2005.07.007 16087353

[CR96] Straube, T., Mentzel, H.-J., & Miltner, W. H. R. (2006b). Neural mechanisms of automatic and direct processing of phobogenic stimuli in specific phobia. *Biological Psychiatry,**59*(2), 162–170. 10.1016/j.biopsych.2005.06.013 16139812

[CR97] Strawn, J. R., Hamm, L., Fitzgerald, D. A., Fitzgerald, K. D., Monk, C. S., & Phan, K. L. (2015). Neurostructural abnormalities in pediatric anxiety disorders. *Journal of Anxiety Disorders,**32*, 81–88. 10.1016/j.janxdis.2015.03.004 PMC4439332 25890287

[CR98] Uddin, L. Q., Yeo, B. T. T., & Spreng, R. N. (2019). Towards a universal taxonomy of macro-scale functional human brain networks. *Brain Topography,**32*(6), 926–942. 10.1007/s10548-019-00744-6 PMC7325607 31707621

[CR99] Vai, B., Parenti, L., Bollettini, I., Cara, C., Verga, C., Melloni, E., Mazza, E., Poletti, S., Colombo, C., & Benedetti, F. (2020). Predicting differential diagnosis between bipolar and unipolar depression with multiple kernel learning on multimodal structural neuroimaging. *European Neuropsychopharmacology,**34*, 28–38. 10.1016/j.euroneuro.2020.03.008 32238313

[CR100] Viña, C., Herrero, M., Rivero, F., Álvarez-Pérez, Y., Fumero, A., Bethencourt, J. M., Pitti, C., & Peñate, W. (2020). Changes in brain activity associated with cognitive-behavioral exposure therapy for specific phobias: Searching for underlying mechanisms. *Revista De Neurología,**71*, 391–398. 10.33588/rn.7111.2019487 33205385

[CR101] Vytal, K., & Hamann, S. (2010). Neuroimaging support for discrete neural correlates of basic emotions: A voxel-based meta-analysis. *Journal of Cognitive Neuroscience,**22*(12), 2864–2885. 10.1162/jocn.2009.21366 19929758

[CR102] Wright, C. I., Martis, B., McMullin, K., Shin, L. M., & Rauch, S. L. (2003). Amygdala and insular responses to emotionally valenced human faces in small animal specific phobia. *Biological Psychiatry,**54*(10), 1067–1076. 10.1016/S0006-3223(03)00548-1 14625149

[CR103] Yamashita, A., Yahata, N., Itahashi, T., Lisi, G., Yamada, T., Ichikawa, N., Takamura, M., Yoshihara, Y., Kunimatsu, A., Okada, N., Yamagata, H., Matsuo, K., Hashimoto, R., Okada, G., Sakai, Y., Morimoto, J., Narumoto, J., Shimada, Y., Kasai, K., Kato, N.,Takahashi, H., Okamoto, Y., Tanaka, S. C., Kawato, M., Yamashita, O., Imamizu, H. (2019) Harmonization of resting-state functional MRI data across multiple imaging sites via the separation of site differences into sampling bias and measurement bias. *PLoS Biol*, *17*(4):e3000042. 10.1371/journal.pbio.3000042 PMC6472734 30998673

[CR104] Yassa, M. A., & Stark, C. E. L. (2009). A quantitative evaluation of cross-participant registration techniques for MRI studies of the medial temporal lobe. *NeuroImage,**44*, 319–327. 10.1016/j.neuroimage.2008.09.016 18929669

[CR105] Yeo, B. T., Krienen, F. M., Sepulcre, J., Sabuncu, M. R., Lashkari, D., Hollinshead, M., Roffman, J. L., Smoller, J. W., Zöllei, L., Polimeni, J. R., Fischl, B., Liu, H., & Buckner, R. L. (2011). The organization of the human cerebral cortex estimated by intrinsic functional connectivity. *Journal of Neurophysiology,**106*(3), 1125–1165. 10.1152/jn.00338.2011 PMC3174820 21653723

[CR106] Xu, L., Groth, K. M., Pearlson, G., Schretlen, D. J., & Calhoun, V. D. (2009). Source-based morphometry: The use of independent component analysis to identify gray matter differences with application to schizophrenia. *Human Brain Mapping,**30*, 711–724. 10.1002/hbm.20540 PMC2751641 18266214

